# Editorial: Characterization of biostimulants used in agriculture: A step towards sustainable and safe foods

**DOI:** 10.3389/fpls.2022.1065879

**Published:** 2022-12-06

**Authors:** Giuseppe Mannino, Cinzia M. Bertea, Paolo Bonini

**Affiliations:** ^1^ Department of Life Sciences and Systems Biology, University of Turin, Turin, Italy; ^2^ oloBion—OMICS LIFE LAB, Barcelona, Spain

**Keywords:** humic substances (HS), protein hydrolysates, arbuscular mycorrhiza (AM), plant growth promoting rhizobacteria (PGPR), macroalgae, microalgae

Over the past decade, sustainable agriculture has experimented the use of biostimulant formulations with the goal of increasing plant resilience to various abiotic stressors, including those related to climate change, such as excessive heat, lack of water, and increased soil salinity (Li et al.). According to the most common and recent definition, biostimulants are formulations that may contain pure molecules or a mixture of bioactive compounds, individual strains or consortia of microorganisms that, when properly applied to plants, lead to an increased agronomic performance by influencing various biochemical and biomolecular pathways (Agliassa et al., 2021). As a result, these formulations are proposed as a viable alternative to the use of fertilizers or other agrochemicals, which can drastically cause additional environmental damage (Rouphael and Colla, 2020).

Although plant biostimulants are now strictly regulated by the most recent legislation 1009/2019, in the past it was unclear how exactly to define these formulations, and specifically what to include in this product category. For example, it should be considered that the definition of plant biostimulant has changed drastically several times over time. Initially, they were defined as formulations able to promote plant growth even when applied in minimal amounts (Rouphael and Colla, 2020). According to this definition, although nutrients and soil conditioners were proven to be effective in promoting plant growth, they were excluded because of the larger amounts needed to achieve a biostimulant effect. Furthermore, according to this definition, a formulation could be labeled as biostimulant if the mechanism of action was closely related to the hormonal one. A more inclusive definition was later coined by du Jardin, who in his study identified eight different categories of formulations: humic substances, complex organic materials (obtained from agro-industrial wastes, slurry extracts, compost, etc.), beneficial chemical elements (Al, Co, Na, Se and Si), inorganic salts, algal extracts (brown, red and green macroalgae), chitin and chitosan derivatives, antiperspirants (kaolin and polyacrylamide), free amino acids and nitrogen-containing substances (peptides, polyamines and betaines) (du Jardin, 2012). However, even according to this classification, microbial biostimulants were still excluded. A new definition of plant biostimulant was again proposed by du Jardin (2015) three years later, in a special issue entitled “Biostimulants in Horticulture’ and conducted by Colla and Rouphael (2015) in Scientia Horticulturae (Colla and Rouphael, 2015). Here, supported by scientific evidences demonstrating the positive action of microbial-based biostimulants, du Jardin suggested the following definition: “*A plant biostimulant is any substance or microorganism applied to plants with the aim to enhance nutrition efficiency, abiotic stress tolerance and/or crop quality traits, regardless of its nutrient content*” (du Jardin, 2015). Today, this definition could be further refined by including among plant biostimulants not only formulations having microorganisms, but also products simply containing bacterial metabolism-derived compounds without containing microbes. Currently, the formulations based on microorganisms or on molecules derived from bacterial metabolism appear to be widely investigated in the literature and have a broad spectrum of action in sustainable agriculture (Castiglione et al., 2021; Ganugi et al., 2021).

Among microbial-based biostimulants, those composed of *Bacillus* spp. are the most commonly used. However, the role and ways through which these microorganisms influence the resilience of plants to abiotic stresses strictly depends on their metabolic capacities (Ganugi et al., 2021). In the research topic “Characterization of Biostimulants Used in Agriculture: A Step Towards Sustainable and Safe Foods,” Nephali et al., demonstrated that the metabolome of *Bacillus* spp. is critical for generating a background essential for biostimulant industries for a more safely and innovatively design of new PGPR-based formulations and decided to combine liquid chromatography coupled with tandem mass spectrometry (LC-MS/MS) with a molecular network (MN) approach, decoding and characterizing the chemical space of four *Bacillus* isolates and a consortium of *Bacillus* strains. The paper shows a differentiated and varied composition of the molecules contained in the four *Bacillus* strains, both when grown separately and/or as a consortium. The MolNetEnhancer networks also identified eleven different molecular families, including lipids and/or lipid-like molecules, benzenoids, nucleotide-like molecules, and organic acid derivatives.

Differently, Bellotti et al., submitted to the Research Topic an article titled “Agronomical valorization of eluates from the industrial production of microorganisms: Chemical, microbiological, and ecotoxicological assessment of a novel putative biostimulant,” in which they evaluate the biostimulatory effect of eluates obtained from culture media industrially used for lactic acid bacteria (LAB) production. Specifically, at industrial level, LABs are grown on a medium rich in macronutrients and micronutrients, and when they reach a sufficient volume are isolated, sold, and employed for human or animal food processing (Erginkaya and Konuray-Altun, 2022). In this process, the remaining growing medium is classified as industrial waste and handed over to companies for its disposal, but this part is extremely costly (Song et al., 2022). However, this eluate represents not only a potential prebiotic formulation for soil microorganisms at the rhizosphere level, but also contains several bioactive compounds capable of inducing interesting physiological responses in plants (Raman et al., 2022). Following the application of eluate in an appropriate formulation, the authors did not record toxic effects on soil, while a positive impact on bacterial diversity was observed. In particular, the authors highlighted a dose-dependent effect, visible even at very low concentrations. However, a general reduction in bacterial diversity was observable with increasing concentration of the by-product, although the relative abundance of some beneficial PGPR species was positively driven by the application of the eluate. Finally, the biostimulant formulation designed by the authors demonstrated the ability to increase the presence of beneficial fungi, while negatively affected the presence of the plant pathogen *Fusarium* spp.

Unlike the previous work, Baghdadi et al. evaluated the biostimulatory effect of another important category of biostimulants, namely a formulation based on *Ascophyllum nodosum* extract. In the past, algae-based extracts have been widely investigated on annual plants, highlighting the potential beneficial action especially on plant morphological, nutritional, nutraceutical traits (Di Stasio et al., 2018; Rouphael et al., 2018; Gonçalves et al., 2020), plant physiological responses (Colla et al.; Goñi et al., 2018; Campobenedetto et al., 2021; Repke et al., 2022), and agronomic yields (Taskos et al., 2019; Ikuyinminu et al., 2022). In the manuscript submitted by Ali Baghdadi to this Research Topic, a robust and comprehensive experimental trial was presented. Indeed, the effects derived from the application of the same biostimulant was evaluated on two different tomato varieties (Micro-Tom and Rio Grande) grown under three environmental conditions (controlled conditions, greenhouse, or field). Surprisingly, the comparison of morpho-physiological and molecular data collected under the different experimental conditions showed consistent results, demonstrating a beneficial effect not only on plant morphology, plant physiology, but also on agronomic yields. Based on these robust and strong results, the authors strongly suggest the combination of transcriptomic and phenomics approaches as a key system to analyze plant responses to any biostimulant.

The collection of these articles in the current Research Topic demonstrates that positive effects on fruit yield and quality can be achieved even without resorting to genetic combination. However, in the manuscript submitted by Priatama et al. a genetic combination of *lpa1* with the semi-dwarf mutant was conducted. The authors’ goal was to create mutants able to reduce water use in agriculture with the aim to produce more environmentally sustainable rice. However, *LPA1* expressed in leaf primordia pre-vascular cells regulates genes associated with carbohydrate metabolism and cell enlargement, thus playing a key role in enlarging of the metaxylem of aerial organs. The narrow metaxylem of *lpa1* shows leaves curling on sunny days and conveys drought tolerance, but reduces grain yield in mature plants. However, genetic combination of *lpa1* with the semi-dwarf mutant (*dep1-ko* or *d2*) provides optimal water supply and drought resistance without compromising grain yield. These results demonstrate that water use and transport can be genetically controlled by optimizing metaxylem vessel size and plant height, which can be used to improve drought tolerance and offers a potential solution for dealing with more frequent adverse weather conditions in the future.

This research theme has included a number of contributions covering different aspects of plant biostimulants. The contributions provide insights that help to understand the modes of action of some biostimulants, and the results presented in this special issue support the design and development of new plant biostimulant prototypes. However, the mechanisms of action through which these formulations exert their positive function have been found to be very heterogeneous, and other scenarios are almost certainly unknown and need to be better studied. Consequently, it is important that research in this area continue focusing not only on the chemical and microbial profile of such formulations, but also on the identification and characterization of their mechanism of action.
